# Diagnostic Role of Plasma MicroRNA-21 in Stable and Unstable Angina Patients and Association with Aging

**DOI:** 10.1155/2020/9093151

**Published:** 2020-04-13

**Authors:** Md Sayed Ali Sheikh

**Affiliations:** Department of Internal Medicine, Cardiology, College of Medicine, Jouf University, Sakaka, Aljouf, Saudi Arabia

## Abstract

The present study explored the clinical value of plasma microRNA-21 as a novel biomarker for early prediction of stable and unstable angina patients and its relationship with aging. A total of 255 participants, 123 patients with chronic stable angina, 82 patients with unstable angina, and 50 healthy subjects, were included in our study. Stable coronary and unstable coronary patients were confirmed following AHA/ACC clinical protocols. Total RNA was extracted from plasma by using miRNA-based TRIzol reagent. Plasma miR-21 expression levels were determined by real-time polymerase chain reaction. To evaluate the diagnosis accuracy, the receiver operating characteristic (ROC) curves were used. Plasma microRNA-21 concentration levels were significantly elevated in stable and unstable angina patients as compared with control subjects (*P* < 0.001). The area under the ROC curves of circulating microRNA-21 was accurately distinguished in stable angina patients (AUC 0.921) and unstable angina patients (AUC 0.944) from healthy subjects. MicroRNA-21 expression gradually elevated with increasing aging in all the populations. Moreover, the current study also demonstrated that the expression of plasma miR-21 levels was significantly associated with different age groups within healthy subjects and stable and unstable angina patients (*P* < 0.001). This research finding suggested that plasma microRNA-21 may be considered as a suitable new biomarker for early detection of stable and unstable angina patients, and it has a strong correlation with aging.

## 1. Introduction

Coronary artery disease (CAD) is a major public health problem and remains the leading cause of sudden cardiac death (SCD) all over the world. Atherosclerosis and older age are the two important risk factors for coronary artery disease. Every year, several millions of chest pain patients were admitted into the hospital in both developed and developing countries. Almost 50% of these chest pain cases were of cardiac origin; either stable angina pectoris, unstable angina, acute coronary syndrome, or acute myocardial infarction (AMI) [1].

An early clinical detection and accurate diagnosis of CAD is an important task for physicians to initiate appropriate treatment and subsequently prevent sudden cardiac death. Although coronary angiography (CAG) is the gold standard invasive test for the diagnosis of CAD patients, it has some limitations including high cost, limited availability, and radiation hazards [2]. Therefore, it is a clinical demand to find out new blood-based biomarkers for identification of CAD in the earlier stage.

Microribonucleic acids (miRNAs) are small (≈22 nucleotides), highly specific, endogenous single-stranded, and noncoding ribonucleic acid (RNA) molecules that regulate gene expression. Recent several research groups evidenced that microRNAs have significant impact on various cardiovascular biology and progression of diseases including coronary artery disease (CAD) and atherothrombosis. MicroRNAs which are detectable in various body fluids are defined as circulating microRNAs [3–5].

It has been demonstrated that expressions of several specific circulating microRNA levels were significantly altered in stable angina pectoris patients (miR-19a, miR-133a, miR-149, and miR-208a), unstable coronary artery disease (miR-423, miR-424, and miR-765), and acute coronary syndrome (miR-1, miR-92a, miR-134-5p, and miR-183-5p). Moreover, plasma miR-208b, miR-499, and miR-223 could be useful as ideal biomarkers for early evaluation of AMI patients [6–12].

Besides, miR-21 has currently received great attention regarding its chronic inflammatory function in coronary atherosclerotic heart disease. Overexpression of miR-21 level significantly promoted abnormal proliferation and migration of vascular smooth muscle cells (VSMCs) and activated the Akt/ERK signaling pathway and aggravated atherosclerosis in a rat model, while knocking down of miR-21 can suppress the activation of VSMCs and reduce atherosclerosis level [13].

Moreover, microRNA-21 expressions in naive CD4+ T cells were amazingly elevated in (65–85 years) older healthy subjects compared with (20–35 years) younger subjects, with higher variance in the geriatric subjects, suggesting miR-21 remarkably controlled immune response and aging [14]. Very recently, it was reported that the expression pattern of circulating microRNA-21 was greatly associated with significant or insignificant coronary stenosis patients [15]. However, the clinical significance of plasma microRNA-21 for coronary artery disease patients and linked with aging is not fully explored. Therefore, the current study investigated the diagnostic potential of circulating microRNA-21 for early detection of stable angina and unstable angina patients and the relationship between plasma miR-21 and aging.

## 2. Materials and Methods

### 2.1. Selection of Study Groups

The current study included 255 participants; among them, 123 patients with chronic stable angina and 82 patients with unstable angina were admitted into the cardiology department of Xiangya Hospital and 50 healthy controls from Xiangya health center from March 2016 to August 2017. Chronic stable coronary angina patients (≥50% blocked in one or more than one major coronary artery) were confirmed by invasive coronary angiography. Moreover, stable angina pectoris and unstable angina patients were characterized by following clinical guidelines of ACC/AHA [16, 17]. However, study patients with less than 28 years and more than 84 years old, chronic inflammatory diseases, autoimmune diseases, acute infectious diseases, neoplastic diseases, chronic liver and kidney diseases (creatinine clearance <15 ml/min), implanted coronary stent, prior acute heart attack, and cardiac failure were not enrolled in this research. Healthy controls were well matched with age, gender, and other basic information with patients' group and also free from cardiovascular and any chronic disease.

This study has been followed all the principles outlined by the revised Helsinki Declaration in 2013 for human subjects. All the study groups have given their written informed consent during recruitment to our study. The Ethical Committee of Xiangya Hospital, Central South University (China), approved this study.

### 2.2. Collection of Blood Samples and RNA Extraction

Cell-and platelet-free plasma were obtained by two-stage centrifugation protocols. Firstly, erythrocytes and other waste products were cleaned by centrifuge at 4°C for 10 min at 1,900 RMP. Secondly, to remove cryoprecipitates and collect fresh plasma, samples were recentrifuged for 10 min at 16,000 RMP at 4°C temperature. After that, all plasma samples were transferred into EP-microtubes and preserved at −80°C. By using TRIzol total RNA extraction reagent, RNA was isolated from plasma following manufactures' guidelines (Invitrogen, Carlsbad, CA, USA). In brief, 250 *μ*L of plasma was mixed with 750 *μ*L of the TRIzol reagent, incubated at room temperature for 5 min, and then mixed with 200 *μ*L chloroform. Then, by centrifuge at 12,000 RMP for 15 min at 4°C, only the upper aqueous phase was collected, and subsequently, RNA was precipitated with adding 500 *μ*L of pure isopropanol. After that, 500 *μ*L of 80% ethanol was added into the RNA samples and cleaned 3 times, and later, RNA samples were diluted with 30 *μ*L DEPC water and kept at 4°C for 10 hours; subsequently, RNA samples' quality and concentration were demonstrated by NanoDrop photometer and stored at −80°C.

### 2.3. Expression of miR-21 Analysis

Expressions of plasma miR-21 were determined by real-time quantitative reverse-transcription polymerase chain reaction (qRT-PCR). RNA sample was reverse-transcribed to cDNA using a specific RT primer, RiboBio reagent of microRNA-21, with an RT-PCR system according to manufacturer's instructions. Real-time quantitative PCR analysis was carried out with a 7300 Real-Time PCR System (Applied Biosystems, CA, USA) by using Takara qRT-PCR primer synthesis kits followed by the company guidelines. Real-time PCR reactions were performed in triplicate for all the study subjects. MiR-156a was used as the internal control to normalize miR-21 expression. Ct values were measured with SDS 2.3 software. The relative expression of microRNA-21 was calculated through the 2^−ΔΔCT^ method (ΔCt microR-21–ΔCt control gene). Those miRNA expressions more than >35 Ct values were not included in this study.

### 2.4. Analysis of Biochemical Parameters

Fasting glucose, C-reactive protein, total cholesterol, triglycerides, high-density cholesterol, low-density cholesterol, and creatinine levels were investigated with commercial kits. Creatine kinase MB and cardiac troponin I concentrations were measured through Beckman immunoassay.

### 2.5. Statistics

Statistical analysis was performed with SPSS version 20.0 software, and graphs were generated using GraphPad Prism 6 version software. Student's *t*-test, one-way ANOVA, Mann–Whitney rank test, Kruskal–Wallis test, Dunn's test, and Fischer's exact test or the chi-square test were used for data analysis as appropriate. The specificity and sensitivity of plasma microRNA-21 for diagnosis of stable and unstable CAD patients were measured through the receiver operating characteristic (ROC) analysis. Differences among the groups were considered statistically significant with *P* < 0.05.

## 3. Results

### 3.1. Clinical Information of the Participants

In total, this study included 123 stable angina patients, 82 unstable angina patients, and 50 control subjects. We compared basic and clinical characteristic among different groups. Male and female, age, smoking, hypertension, type-2 DM, HR, SBP, DBP, and EF were not statistically significant (*P* > 0.05). Moreover, family history of CHD and C-reactive protein level between controls with stable and unstable angina patients were highly significant (*P* < 0.001). The detailed information is shown in Table 1.

### 3.2. Plasma miR-21 Concentration Level in Stable and Unstable Angina Patients and Control Subjects

**Figure 1:**
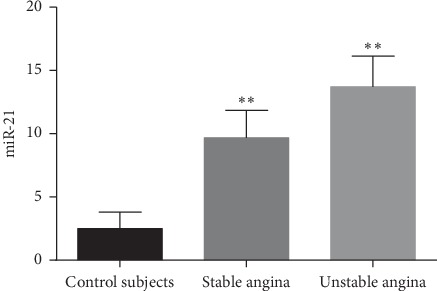
The expression of plasma miR-21 in stable CAD, unstable CAD, and control subjects. Control subjects vs. stable CAD (*P* < 0.001); control subjects vs. unstable stable CAD (*P* < 0.001).

This study investigated the plasma miR-21 concentration level from stable angina patients, unstable angina patients, and control subjects (Figure 1). We found that plasma miR-21 concentrations were remarkably upregulated in patients with stable angina and unstable angina groups than healthy control groups (*P* < 0.001). Moreover, the expressions of plasma miR-21 level in unstable angina patients were relatively higher than stable angina patients, but the differences were not statistically significant (*P* > 0.05).

### 3.3. Diagnostic Significance of Plasma MicroRNA-21 Level in Stable and Unstable Angina Patients

**Figure 2:**
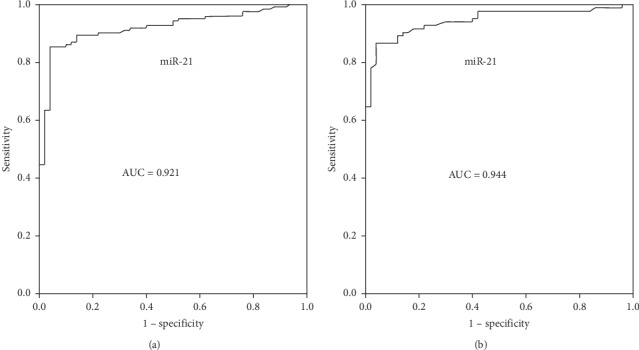
ROC curve analysis to estimate the diagnostic significance of plasma microRNA-21 in stable and unstable CAD patients. (a) Control subjects and stable angina patients (AUC 0.921). (b) Control subjects and unstable angina patients CAD (AUC 0.944).

Diagnostic accuracy of circulating micoRNA-21 was evaluated through ROC curve analyses. Plasma microRNA-21 was able to strongly separate stable angina patients (AUC 0.921) and unstable angina patients (AUC 0.944) from healthy control subjects (Figures 2(a) and 2(b)). These findings recommended that plasma concentrations of microRNA-21 may be a useful biochemical marker for diagnosis of CAD patients.

### 3.4. Relationship of miR-21 with Aging

**Figure 3:**
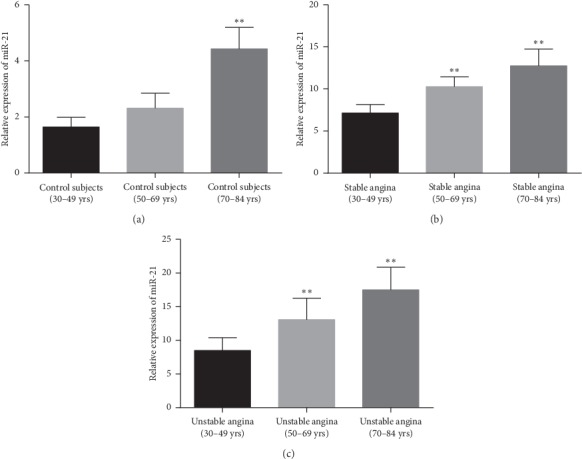
(a) Expression of plasma miR-21 among different age groups within control subjects. (b) Plasma microRNA-21 concentrations within stable angina patients in different age groups. (c) Comparison of circulating microRNA-21 levels within unstable angina patients in variation of ages.

The current study measured circulating miR-21 expression at different age variations among all the study subjects. Circulating microRNA-21 expression gradually elevated with increasing age in all the populations. Plasma microRNA-21 levels were evidently associated with different age groups within healthy subjects and stable and unstable angina patients (*P* < 0.001) (Figure 3).

## 4. Discussion

The present study supported the prospective clinical value of cardiac specific plasma miR-21 as an important blood-based biomarker for early detection of stable and unstable angina pectoris patients. The current study results showed that plasma microRNA-21 was barely detectable in healthy control subjects, but microRNA-21 level was significantly elevated in both stable and unstable angina patients. These results are also in agreement with other research findings in coronary artery disease patients [18, 19]. Moreover, this research investigated the diagnostic importance of plasma miR-21 with ROC analysis. The current study found that AUC values for miR-21 were remarkably increased in stable angina patients (0.921) and unstable angina patients (0.944) with high specificity and sensitivity compared with healthy controls. Taken together, these research results demonstrated that plasma microRNA-21 might be a potential biochemical marker for diagnosis of stable and unstable ischemic heart disease patients.

Sanlialp et al. reported miR-21 expression levels in patients with coronary artery disease were significantly higher than control subjects [20]. A very recent research also demonstrated circulatory microRNA-21 in stable CAD patients was upregulated by 1.90 fold, and AUC was 0.79 and with high sensitivity and specificity and suggested it is a possible diagnostic marker for coronary heart disease [21].

It has been well established that aging is a worldwide problem affecting both developed and developing countries. Aging is a multifactorial process characterized by gradually decrease in physiological and biochemical activities of individual tissues and organs. Geriatric age is the fixed nonmodifiable major contributor for atherosclerotic coronary artery disease. Circulating miR-21 can modulate human gene expression and regulate immune system response and directly link to atherosclerosis and aging. Rusanova et al. demonstrated that miR-21 had higher expression in geriatric people than young adults. In addition, expression of plasma cytokine (IL-6, IL-8, IL-10, and TNF*α*) levels was increased in aged group people (>71 yrs old) compared with young control groups (>18 yrs old). Furthermore, they also found that expression of miR-21, plasma-advanced oxidation protein products (AOPP), and the TNF*α*/IL-10 ratio were positively correlated in elderly patients compared with younger [22].

Recently, Ahmed et al. revealed that miR-21 levels were prominently increased in both aged mouse (>2 yrs old) and human aged (>78 yrs old) skin as compared with control groups through negative regulation of chromatin remodeller special-AT-rich-sequence-binding protein-1 (SATB1) in keratinocytes and suggested miR-21 remarkably contributed to aging [23].

It was previously demonstrated that microRNA-21 is critically involved in the regulation of different inflammatory pathways and development of atherosclerosis. Moreover, very recently, it was reported that expression of serum miR-21 levels was notably elevated in atherosclerotic subjects and stroke patients relative to healthy individuals. Likewise, they also found that miR-21 strongly regulated the anti-inflammatory effect of haem oxygenase-1 (HO-1) in aged intracerebral hemorrhagic rats [24]. Besides, plasma microRNA-21 was also significantly correlated with platelet extracellular vesicles (EVs) in stable coronary artery disease patients [25].

Darabi et al. found that circulating miR-21 levels and matrix metalloproteinase-9 (MMP-9) were significantly higher in acute coronary syndrome (ACS) patients compared with stable coronary artery disease patients. Additionally, expression of miR-21 levels was also positively correlated with MMP-9, high sensitivity C-reactive protein, and aging. The present study also found that C-reactive protein and history of coronary artery disease were significantly higher in stable and unstable angina patients than controls [26].

However, for the first time, this research examined that expression of the plasma miR-21 level was significantly higher in (70–84 years) elderly people as compared with 30–49 years group people, suggesting elevated circulating miR-21 level may be helpful for diagnosis of age-related coronary atherosclerosis.

To decline possible bias from the patient selection, subjects with less than 30 years or more than 84 years were excluded from our study, and the male/female ratio was well balanced between coronary artery disease and healthy controls. However, to minimize the coronary angiography catheter and dye-induced effect on endothelial injury and blood circulation as well as expression of microRNA-21, in this study, all the blood samples were collected from the CAD patients after 72 hours of coronary angiography.

Furthermore, the present study carefully recorded basic data, risk factors, and laboratory information such as HR, SBP, DBP, EF, current smoking, hypertension, hyperlipidemia, type-2 DM, family history of CHD, fasting glucose, C-reactive protein, lipid profiles, creatinine, creatine kinase MB, and troponin I. Statistical analysis demonstrated that clinical characteristics among healthy groups and coronary artery disease patients were not influenced by miR-21 level in plasma, suggesting miR-21 as a potential biochemical biomarker for diagnosis of stable and unstable angina patients.

To avoid possible confounders derived from qRT-PCR methods, the present study used miR-156a as an inner control as compared with other available synthetic endogenous miRNAs because in our previous studies and also in other studies, it was confirmed that miR-156a is highly stable [7, 27, 28]. Furthermore, this study examined every sample in four times; Ct values of miR-21 from 15–35 were added during analysis which strongly suggested these findings are more accurate and reliable.

However, the current research is based on a single center and comparatively smaller size population. Also, this study did not investigate the pathway of the underline molecular mechanism of miR-21 linked with coronary artery disease and aging. Future studies are required to confirm the clinical value and pathological significance of this biomarker.

## 5. Conclusion

High level of plasma miR-21 has potential utility as a novel blood-based biomarker for clinical diagnosis of stable and unstable angina patients. Moreover, we also recommended that miR-21 has a strong correlation with aging.

## Figures and Tables

**Table 1:** Clinical information of the participants.

Characteristics	Control subjects (*n* = 50)	Stable angina (*n* = 82)	Unstable angina (*n* = 50)	*P*_1_	*P*_2_	*P*_3_
Gender, male/female (*n*/*n*)	30/20	58/24	32/18	0.408	0.671	0.439
Age (yrs)	59.9 ± 7.2	63.7 ± 11.4	65.08 ± 9.1	0.270	0.536	0.331
HR(bpm)	71.02 ± 6.4	74 ± 5.3	75 ± 9.2	0.825	0.798	0.811
SBP (mmHg)	124.9 ± 5.41	135.6 ± 9.25	139.±13.14	0.190	0.476	0.607
DBP (mmHg)	74.8 ± 8.13	77.48 ± 10.25	80.71 ± 9.21	0.731	0.332	0.395
EF (%)	63.4 ± 8.7	59.4 ± 6.05	56 ± 7.21	0.075	0.064	0.332
*Risk factors, n (%)*						
Current smoking	31 (62)	52 (64)	34 (67)	0.362	0.135	0.544
Hypertension	30 (59)	56 (68)	36 (72)	0.459	0.217	0.709
Hyperlipidemia	17 (33)	39 (48)	27 (54)	0.135	0.082	0.268
Type-2 DM	11 (22)	24 (29)	17 (33)	0.784	0.355	0.759
Family history of CHD	6 (11)	34 (41)	22 (43)	<0.001	<0.001	0.812
*Laboratory information*						
Fasting glucose (mmol/L)	4.5 ± 0.9	5.34 ± 1.1	5.54 ± 1.1	0.267	0.218	0.530
C-reactive protein (mg/L)	1.2 ± .0.5	8.4 ± 1.5	11.5 ± 7.8	<0.001	<0.001	0.085
Total cholesterol (mmol/L)	4.21 ± 1.1	5.1 ± 1.6	5.48 ± 1.1	0.580	0.466	0.749
Total glyceride (mmol/L)	1.5 ± 0.9	1.63 ± 0.8	1.77 ± 1.6	0.372	0.075	0.214
High-density lipoprotein (mmol/L)	1.41 ± 0.2	1.12 ± 0.2	1.04 ± 0.6	0.628	0.431	0.429
Low-density lipoprotein (mmol/L)	2.9 ± 1.04	3.41 ± 1.8	3.62 ± 1.5	0.221	0.138	0.632
Creatinine (umol/L)	81.7 ± 16.1	84.4 ± 31.1	88.3 ± 42.2	0.536	0.459	0.846
Creatine kinase MB	0	9.08 ± 3.5	11.2 ± 1.7			
Troponin I	0	0.02 ± 0.01	0.04 ± 0.06			

Data are presented as mean ± SD. Abbreviations: HR, heart rate; bpm, beats/minute; SBP, systolic blood pressure; DBP, diastolic blood pressure; EF, ejection fraction; type-2 DM, diabetes mellitus; CHD, coronary heart disease; *P*_1_ (controls and stable angina), *P*_2_ (controls and unstable angina), and *P*_3_ (stable angina and unstable angina).

## Data Availability

Data used for this current study are accessible on reasonable request from the corresponding author.
